# Introducing navigation during melanoma-related sentinel lymph node procedures in the head-and-neck region

**DOI:** 10.1186/s13550-017-0312-1

**Published:** 2017-08-17

**Authors:** Gijs H. KleinJan, Baris Karakullukçu, W. Martin C. Klop, Thijs Engelen, Nynke S. van den Berg, Fijs W. B. van Leeuwen

**Affiliations:** 1 Interventional Molecular Imaging Laboratory, Department of Radiology, Leiden University Medical Hospital, Albinusdreef 2, C2-S zone, 9600, 2300 RC Leiden, the Netherlands; 2 Department of Nuclear Medicine, The Netherlands Cancer Institute–Antoni van Leeuwenhoek Hospital, Plesmanlaan 121, 1066 CX Amsterdam, the Netherlands; 3 Department of Head and Neck Oncology, The Netherlands Cancer Institute–Antoni van Leeuwenhoek Hospital, Plesmanlaan 121, 1066 CX Amsterdam, the Netherlands

**Keywords:** Virtual reality, Augmented reality, Surgical navigation, Sentinel node, Melanoma, Fluorescence, Dual modality imaging, Nuclear medicine, Image guided surgery

## Abstract

**Background:**

Intraoperative sentinel node (SN) identification in patients with head-and-neck malignancies can be challenging due to unexpected drainage patterns and anatomical complexity. Here, intraoperative navigation-based guidance technologies may provide outcome. In this study, gamma camera-based freehandSPECT was evaluated in combination with the hybrid tracer ICG-^99m^Tc-nanocolloid.

**Materials and methods:**

Eight patients with melanoma located in the head-and-neck area were included. Indocyanine green (ICG)-^99m^Tc-nanocolloid was injected preoperatively, whereafter lymphoscintigraphy and SPECT/CT imaging were performed in order to define the location of the SN(s). FreehandSPECT scans were generated in the operation room using a portable gamma camera. For lesion localization during surgery, freehandSPECT scans were projected in an augmented reality video-view that was used to spatially position a gamma-ray detection probe. Intraoperative fluorescence imaging was used to confirm the accuracy of the navigation-based approach and identify the exact location of the SNs.

**Results:**

Preoperatively, 15 SNs were identified, of which 14 were identified using freehandSPECT. Navigation towards these nodes using the freehandSPECT approach was successful in 13 nodes. Fluorescence imaging provided optical confirmation of the navigation accuracy in all patients. In addition, fluorescence imaging allowed for the identification of (clustered) SNs that could not be identified based on navigation alone.

**Conclusions:**

The use of gamma camera-based freehandSPECT aids intraoperative lesion identification and, with that, supports the transition from pre- to intraoperative imaging via augmented reality display and directional guidance.

## Background

Over the past decades, the sentinel node (SN) biopsy procedure for loco-regional lymph node (LN) staging in patients with (head-and-neck) melanoma has increasingly gained interest [1, 2]. This procedure allows preoperative identification of the primary tumor-draining LNs (so-called sentinel nodes (SNs)) using lymphoscintigraphy and single-photon emission computed tomography combined with computed tomography (SPECT/CT) imaging [3]. This information can then be used to provide the basis for a surgical roadmap.

Differences in patient placement during preoperative imaging and head-and-neck surgery complicate the direct translation of the preoperative findings to the surgical field of view. Intraoperative guidance is therefore required in the form of a gamma-ray detection probe (referred to as gamma probe) [4, 5] or portable/handheld gamma cameras that provide a superior sensitivity and high resolution [6, 7]. Both techniques, however, lack in-depth information and features that can be complemented through the use of superficial optical imaging/fluorescence guidance. Conversely, fluorescence imaging is limited by tissue-induced signal attenuation, making the technology dependent on other in-depth imaging technologies such as SPECT.

In order to provide placement of radioactive hotspots into anatomical context, optical and gamma tracing modalities can be physically integrated [8–10]. Alternatively, navigation of surgical tools/modalities in a manner analog to the use of global positioning systems (GPS) instead of an old-fashion paper roadmap can be employed. Navigation was successfully introduced in radioguided surgery via the use of geometrically tracked gamma probes that generate freehandSPECT scans that can be presented as augmented reality views [11–14]. Uniquely, these 3D datasets also allow for surgical navigation by providing dynamic feedback with regard to the distance of the gamma probe to the lesion of interest, e.g., SNs of head-and-neck malignancies [12, 15, 16]. Limiting factors in the practical application of this technology are the sensitivity and the time that is required to generate a freehandSPECT scan. Recently, we presented that, in breast cancer, intraoperative use of a handheld gamma camera rather than a gamma probe for freehandSPECT acquisition could overcome these shortcomings [6]. Others have used this approach for SN biopsy in different malignancies and for the detection of parathyroid adenoma [17–19].

In the current clinical pilot study, the feasibility of the use of a handheld gamma camera for intraoperative freehandSPECT acquisition and subsequent navigation-guided surgery was explored in patients with head-and-neck melanoma. Indocyanine green (ICG)-^99m^Tc-nanocolloid was used to help validate the accuracy of the navigation procedure, as this tracer can be detected using both freehandSPECT and high-resolution fluorescence imaging [20].

## Methods

### Patients

Eight patients with histology-proven melanoma in the head-and-neck area, who were scheduled for wide re-excision of the melanoma scar and a SN biopsy procedure, were included (for patient characteristics see Table 1). Clinically, the regional LNs of the patients were tumor-negative as defined by palpation, ultrasound, and fine-needle aspiration cytology.

**Table 1:** Patient characteristics, preoperative and intraoperative findings, and pathology

				Preoperative findings				Intraoperative findings					Pathology	
	Age	Clinical T-stage	Tumor location	Administered dose (MBq)	No. of SNs on lymphoscintigrams	No. of SNs on SPECT/CT	Location SNs	Total no. of removed SNs	Fluorescent SNs		Radioactive SNs		Total no. of SNs	No. tumor-positive nodes
									In vivo	Ex vivo	In vivo	Ex vivo		
1	60	T2a	Right cheek	82.0	0	4	Parotid gland (2×), level I (2×)	4	3	4	4	4	4	0
2	74	T2b	Left cheek	84.9	3	3	Parotid gland, level II, level V	4	4	4	4	4	6	0
3	67	–	Right eyelid	84.4	1	1	Parotid gland	1	1	1	1	1	1	0
4	52	T3b	Right cheek	78.2	1	1	Level V	1	1	1	1	1	1	0
5	59	T4b	Left occipital region	90.2	1	1	Level V	1	1	1	1	1	1	1
6	63	T3a	Right occipital region	101.4	0	1	Level II	3	3	3	3	3	4	0
7	66	T2a	Vertex	75.5	2	2	Level II (2×)	3	3	3	3	3	4	0
8	43	T3a	Left ear	90.1	2	2	Parotid gland, level II	3^a^	3	3	3	3	3	1
Average	60.5			85.8	1.25	1.9		2.5	2.4	2.5	2.5	2.5	3	
Total					10	15		20	19	20	20	20	24	2

*MBq* megabequerel, *SN* sentinel node, *SPECT/CT* single-photon emission computed tomography combined with computed tomography

^a^Additional SN near injection site found with transcutaneous fluorescence imaging (and confirmed using the gamma probe) which was not seen on preoperative imaging

Prior to the commencement of the study, approval from the institutional review board of The Netherlands Cancer Institute—Antoni van Leeuwenhoek was obtained and patients were only included after written informed consent was provided.

### Preoperative procedure

Preparation and injection of the hybrid tracer ICG-^99m^Tc-nanocolloid, as well as the applied preoperative imaging procedure have previously been described [20]. ICG-^99m^Tc-nanocolloid was injected intradermally in four deposits (0.1 mL/deposit) surrounding the melanoma scar. Lymphoscintigraphy (15 min and 2 h post-injection) and SPECT/CT imaging (2 h post injection) were performed in order to determine the number and location of the SN-related hotspots. For SPECT/CT acquisition, the patient was placed in a supine position, with a straight neck. Preoperative findings are provided in Table 1.

### Intraoperative procedure

#### Reference tracker placement

Placement of reference trackers for acquiring freehandSPECT images and the setup for navigation were carried out according to procedures described by Engelen et al. [6]. In short, after anesthetizing the patient and sterilizing the operation field, the neck of the patient was positioned in such a way that the surgeon had easy access to the SNs on one side of the neck. Thereafter, a sterile reference tracker (referred to as RT_p_) was placed on the skull of the patient, followed by placement of a second reference tracker (referred to as RT_hgc_) onto the handheld gamma camera (ChrystalCam; Chrystal Photonics, Berlin, Germany). Finally, a third reference tracker (referred to as RT_gp_) was placed on the gamma probe (Chrystal probe; Chrystal Photonics).

To ensure continuous capture of all reference trackers in the field of view of the navigation system, the optical tracking system was placed in the direct line-of-sight with the RT_p_, above the head of the patient. Near-infrared optical tracking of the fiducials present on the RT_p_, the RT_hgc_, and the RT_gp,_ the navigation system (declipseSPECT; SurgicEye, Munich, Germany) was used to determine the position and orientation of the patient, the handheld gamma camera, and the gamma probe and to place these features in the same coordinate system [6, 21]. The tip of the gamma probe (approx. 1 cm in diameter) was used for the navigation, as this allowed easier identification of the SNs compared to the use of the bulkier handheld gamma camera.

#### FreehandSPECT acquisition in the head-and-neck area using a handheld gamma camera

The 2D gamma-imaging mode of the handheld gamma camera was used to roughly localize the area harboring the SNs and to center the radioactive hotspot in the volume of interest (VOI; 12 × 12 × 12 cm) of the freehandSPECT. After defining the position of the VOI, the geometrically tracked handheld gamma camera was used to scan the VOI in different directions whereby the declipseSPECT device provided feedback on the radioactive counts collected. When >2500 counts were collected, the acquisition was stopped and the freehandSPECT image reconstructed. Subsequently, the “tracked” gamma probe was navigated by the surgeon until the intact skin was reached. The accuracy of this position was then evaluated by comparing the position of the “tracked” gamma probe with that of a second gamma probe that was placed based on acoustic guidance.

#### Sentinel node identification: navigation, gamma probe and fluorescence guidance

After incision, the SN was pursued using the conventional approach of combined gamma tracing (Neoprobe; Johnson & Johnson Medical, Amersfoort, the Netherlands) and fluorescence imaging (PhotoDynamic Eye (PDE); Hamamatsu Photonics K.K., Hamamatsu, Japan) in a manner similar as described previously [20]. When the SN was visible, the “tracked” gamma probe was navigated towards the SN using the freehandSPECT scan acquired prior to placement of the incision. The distance from the tip of the “tracked” gamma probe to the SN for each procedure, as reported by the navigation device, is provided in Table 2.

**Table 2:** Intraoperative freehandSPECT findings

	Acquisition time (s)	% VOI scanned	Reconstruction time (s)	No. of SNs on freehandSPECT/total no. of SNs preoperative imaging (%)	No. of SNs located with navigation/no. of SNs seen on freehandSPECT (%)	Error of navigation per SN (mm)	Note
1	85	66.3	31	3/4 (75)	3/3 (75)	7, 8, 4	SN in parotid gland not visible on freehandSPECT
2	100	78	n.n.	3/3 (100)	3/3 (100)	9, 9, 9	Level V cluster of 2
3	121	67.6	87	1/1 (100)	1/1 (100)	5	–
4	126	51.5	211	1/1(100)	1/1 (100)	2	–
5	94	71	31	1/1(100)	1/1 (100)	5	–
6	74	59	89	1/1 (100)	0/1 (0)	–	SN part of IS, navigation not possible; level II SN cluster of 3
7	132	82	135	2/2 (100)	2/2 (100)	0, 5	Level II cluster of 2
8	199	80.4	90	2/2 (100)	2/2 (100)	7, 5	–
Average	116.4	69.5	96.3			5.8	
Total				14/15 (93.3)	13/14 (92.9)		

*SN* sentinel node, *SPECT/CT* single-photon emission computed tomography, *VOI* volume of interest, *3D* three-dimensional, *n.n.* not noted, *IS* injection site

A post-excision freehandSPECT was generated after removal of the SNs to evaluate possible residual radioactivity present in the VOI. A mobile gamma camera (Sentinella; Oncovision, Valencia, Spain) was used to confirm removal of the preoperatively identified SNs [22].

The techniques used in this study and the type of information they provide during the surgical procedure are described in Table 3. Since the study entailed the evaluation of a new technology, this resulted in the duplication of gamma-probe and gamma-camera systems.

**Table 3:** Information provided by different intraoperative imaging modalities

	2D information	3D information	Acoustic read-out	Numerical read-out	Visual read-out	Depth information	Anatomical detail
Neoprobe gamma probe^a ,b^	−	−	+	+	−	−	−
Sentinella gamma camera^a, b^	+	−	+	+	+ (Gamma image)	−	−
PDE fluorescence camera^a, b^	+	−	−	−	+ (Fluorescence image)	+/−	+
(Chrystal) gamma camera combined with freehandSPECT (incl. Navigation of Chrystal gamma probe and acoustic confirmation with the same probe)^a^	+	+	+	+	+ (Gamma image)	+	−
Fluorescence camera combined with freehandSPECT	+	+	+	+	+ (Gamma + fluorescence image)	+	+

*2D* two-dimensional, *3D* three-dimensional

^a^Technologies applied in this study

^b^Routine modality used for the procedures describe in the study

### Pathology

Excised SN specimens were formalin-fixed and the nodes present in the specimens counted before being bisected and paraffin-embedded. Tissue sections cut at 50–150-μm intervals were used for histopathological evaluation and evaluation of the presence of nodal metastasis [20].

## Results

### Preoperative imaging procedure

With preoperative lymphoscintigraphy and SPECT/CT imaging, a total of 15 SN-related hotspots were identified (Table 1). Interestingly, in one patient (patient 1) non-visualization occurred on early- and late lymphoscintigrams while with SPECT/CT four SN-related hotspots were identified (Table 1).

Direct translation of the preoperative SPECT/CT scans to the surgical setting was not always possible, due to the difference in patient positioning during the preoperative SPECT/CT scan and the intervention. Complexity of translation further increased when SN-related hotspots were identified on both sides of the neck, which required repositioning of the patient during surgery in order to expose both sides of the neck. These features complicated the surgeon’s ability to relate anatomical reference points in preoperative SPECT/CT to those in the intraoperative situation.

### Pre-incision imaging procedure

On average, freehandSPECT acquisition took a mere 116.4 s (range 74–199 s), in which an average of 69.5% of the VOI was scanned (range 51.5–82.0%). FreehandSPECT reconstruction time was on average 96.3 s (range 31–211 s; Table 2). As the patient was immobilized on the operation table, the acquired freehandSPECT scans were limited by the degree of freedom wherein the camera could be positioned over the lesion in order to generate a 3D image. Furthermore, the limited volume of interest that was scanned (12 × 12 × 12 cm) resulted in the acquisition of multiple freehandSPECT in the first two patients.

**Fig. 1:**
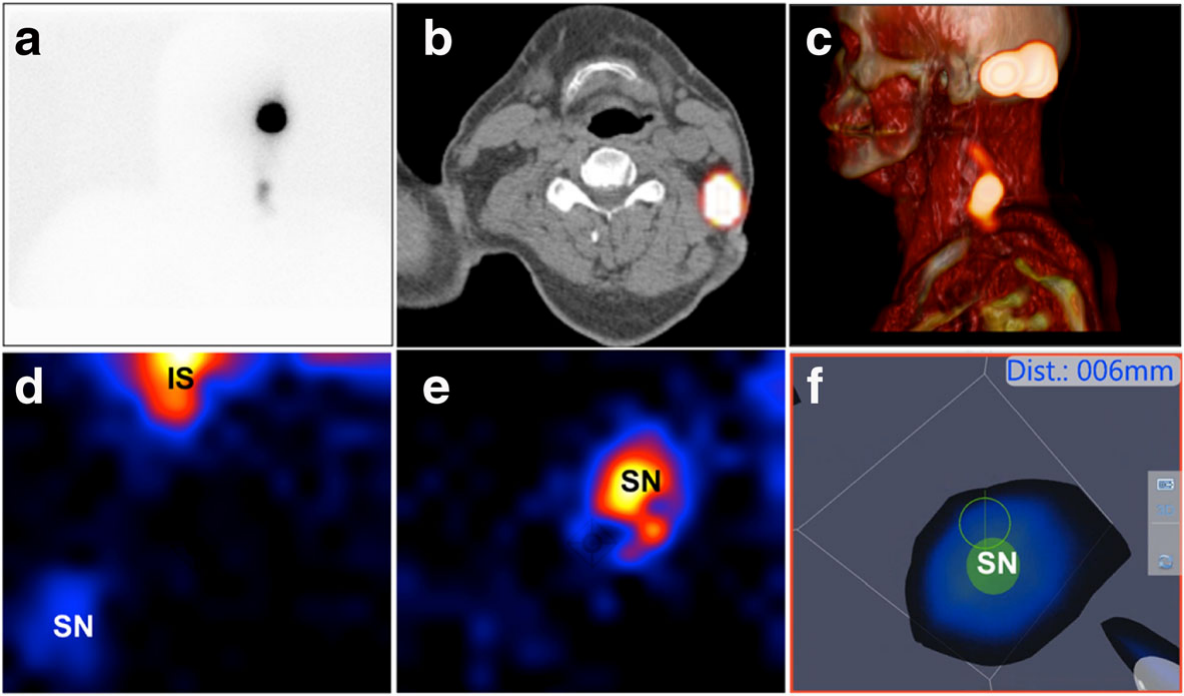
Overview of the acquired images. **a** Example of an anterior lymphoscintigram showing a clear sentinel node in the neck. **b**, **c** SPECT/CT imaging of the patient shown under **a** allowed placement of the hotspot in its anatomical context with the sentinel node being located in level V. The fused SPECT/CT images provided the surgeon with an anatomical roadmap for planning of the surgical procedure. **d** 2D mobile gamma camera image acquired in the operation room showing a sentinel node (SN) and the injection site (IS). **e** Zoom-in of the image shown in **d**. **f** A freehandSPECT scan was acquired and subsequently the gamma probe was navigated, in augmented reality, to the sentinel node as seen in the freehandSPECT scan

Using preoperative SPECT/CT as a reference for identified SNs, intraoperatively obtained freehandSPECT images provided a 93% detection rate (14/15 SN-related hotspots visualized). When identified, the exact location of the SNs in the surgical setup could be depicted as an augmented reality overlay. As demonstrated by a typical example in Fig. 1, handheld gamma camera and freehandSPECT scans depicted the same features as the preoperatively acquired lymphoscintigrams and SPECT/CT images in 75% of patients (no complete conformity in patients 1 and 6).

In patient 1, four SN-related hotspots were preoperatively identified with SPECT/CT. In this patient, low tracer uptake in a SN located in the parotid gland prevented detection using freehandSPECT. In patient 6, a lower-activity SN-related hotspot near the high-activity injection site was identified on preoperative SPECT/CT which could also not be identified using freehandSPECT. A cluster of SN-related hotspots in level II was identified on preoperative SPECT/CT in patient 7, which could be differentiated into three SN-related hotspots after examination of the freehandSPECT scan.

### Post-incision imaging procedure

**Fig. 2:**
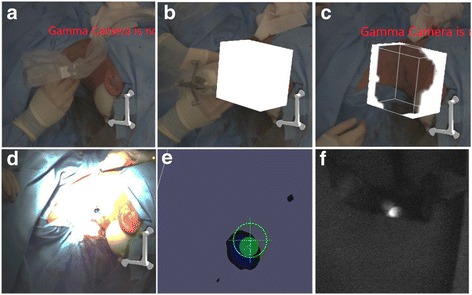
Intraoperative navigation procedure. **a** Pre-navigation overview. **b**, **c** Intraoperative freehandSPECT acquisition. **d** Augmented reality following reconstruction of the acquired data wherein a 3D overlay is obtained. **e** Navigation of the gamma probe in 3D virtual reality. **f** Optical confirmation of sentinel node localization via fluorescence imaging

Placement of the tracker on the rigid skull and outside the surgical field prevented the need for replacement during the surgical procedure and resulted in minimal deformations. The “tracked” gamma probe could be virtually navigated in seven patients (13 of the 14 SN-related hotspots (93%)) with a navigation inaccuracy of 5.8 mm in the numeric distance to the target (Table 2, Figs. 1 and 2). It should be noted that this inaccuracy seemed to be influenced for a large part by the mere 3-mm spatial resolution of the freehandSPECT images [23]. Inaccuracy induced by movement artifacts could be contributed to e.g. the incision process or retractors used. In all cases wherein the navigation procedure was slightly inaccurate, identification of the SNs was enabled by a manual correction based on fluorescence imaging.

In cases wherein the navigation option could not be used, the combined use of the SPECT/CT images, gamma probe, and fluorescence camera allowed identification of the SNs (Table 2). In patients 1 and 6, the superior spatial resolution of fluorescence imaging allowed localization of the SNs that were not detected by freehandSPECT. In patient 6, intraoperative fluorescence imaging revealed three SNs at the location of the hotspot that was identified on SPECT/CT. Post-SN-excision freehandSPECT and the use of the alternative mobile gamma camera confirmed accurate removal of the SNs in all patients.

### Pathology

Pathological evaluation of the biopsy specimens resulted in identification of a total of 24 nodes, of which two were tumor-positive (found in patients 5 and 8; Table 1). In patient 8, a tumor-positive SN was found in the parotid gland, while in patient 5, a tumor-positive node was located in the re-excision specimen of the melanoma scar. This last node was overshadowed by the high-activity of the injection site, which prevented identification on preoperative images and was therefore not explored during the operation.

## Discussion

The results described in this study demonstrate that intraoperative freehandSPECT scans that are generated using a handheld gamma camera provide a 93% detection rate of SNs that were preoperatively identified on SPECT/CT identified in the head-and-neck area. The use of the hybrid tracer ICG-^99m^Tc-nanocolloid allowed for the (high-resolution) detection of the remaining SNs using fluorescence imaging. During the surgical procedure, the freehandSPECT device helped to place the nuclear medicine findings within the anatomical context. In addition, the use of an augmented reality overlay also provided dynamic information with regard to the distance to the target.

Due to the common occurrence of so-called clustered nodes in the head-and-neck area [20], there continues to be a discrepancy between the SN-related hotspots identified at SPECT/CT and the actual number of SNs removed during surgery (38% increase in this particular study; 24 in Table 1 vs. 15 in Table 2). Unfortunately, intraoperative use of freehandSPECT did not demonstrate the resolution and real-time confirmation that is required to solve this issue. Hence, resection of all SNs in one hotspot still demands the use of high-resolution and real-time feedback, as is provided by fluorescence imaging.

When using preoperative SPECT/CT scans for navigation purposes, identical RT_p_ placement in the pre- and intraoperative setting was required to limit the degree of deformation [24–27]. This practical limitation was now overcome by the use of intraoperatively generated freehandSPECT scans. Unfortunately, the relatively small volume of interest of the freehandSPECT (12 × 12 × 12 cm) resulted in the generation of multiple freehandSPECT scans in some patients. The disruption of the surgical workflow was minimized by the prior knowledge of the location wherein the SNs resided. Such disruption, however, remains common during the introduction of new technologies and can be contributed to the early stage development of the technology as well as the limited experience with the technology (learning curve). It may be envisioned that integration of the freehandSPECT and navigation options in the surgical workflow can be optimized further from a technical point of view. For example, prevention of duplication of modalities (see Table 3) would already save time. Based on the fact that surgeons used the depth estimation provided by the navigation setup to estimate the risk of damage to delicate tissues, one may also reason that striking a balance between cure- and surgery-induced toxicity would warrant a slight prolongation of the surgical procedure.

Previously, we demonstrated that preoperative SPECT/CT remains incremental in the SN identification process, even when fluorescence-based surgical guidance to the same target is available [20]. Given the revealed need for positional information during placement of the freehandSPECT VOI, we see no reason to deviate from this point of view. This study, however, does illustrate how freehandSPECT imaging and the matching “GPS-like” navigation capabilities can help strengthen the connection between the findings of both modalities [28]. The use of the hybrid tracer (ICG-^99m^Tc-nanocolloid), a tracer that can be detected using both modalities [24], enabled complementary use of nuclear and fluorescent technologies. In the current study, a gamma probe was used for navigation, but in the future, other tools may be positioned using navigation, for example, a fluorescence camera that displays a real-time augmented reality overlay of freehandSPECT data within the fluorescence images (see Table 3) [26]. In such an integrated image guided surgery approach, the use of augmented reality displays, virtual navigation, and fluorescence guidance can all be used in the same setting [29].

## Conclusions

Generation of an intraoperative freehandSPECT scan using the handheld gamma camera/navigation system allows for the identification of SNs in the head-and-neck area, with an accuracy that approaches that of conventional SPECT/CT. The augmented reality display and directional positioning options provided by the navigation system help refine lesion localization, compared to traditional radioguided surgery tools.
